# Phylogeography, genetic diversity, and population structure of Nile crocodile populations at the fringes of the southern African distribution

**DOI:** 10.1371/journal.pone.0226505

**Published:** 2019-12-23

**Authors:** Barbara van Asch, William F. Versfeld, Kelvin L. Hull, Alison J. Leslie, Timoteus I. Matheus, Petrus C. Beytell, Pierre du Preez, Ruhan Slabbert, Clint Rhode

**Affiliations:** 1 Department of Genetics, Stellenbosch University, Private Bag X1, Matieland, South Africa; 2 Department of Conservation Ecology and Entomology, Stellenbosch University, Private Bag X1, Matieland, South Africa; 3 Directorate of Scientific Services, Department of Natural Resources Management, Ministry of Environment & Tourism, Private Bag 13306, Windhoek, Namibia; 4 Department of Ancient Studies, Stellenbosch University, Private Bag X1, Matieland, South Africa; Senckenberg am Meer Deutsches Zentrum fur Marine Biodiversitatsforschung, GERMANY

## Abstract

Nile crocodiles are apex predators widely distributed in sub-Saharan Africa that have been viewed and managed as a single species. A complex picture of broad and fine-scale phylogeographic patterns that includes the recognition of two species (*Crocodylus niloticus* and *Crocodylus suchus*), and the structuring of populations according to river basins has started to emerge. However, previous studies surveyed a limited number of samples and geographical regions, and large areas of the continent remained unstudied. This work aimed at a fine scale portrait of Nile crocodile populations at the fringes of their geographic distribution in southern Africa. Wild and captive individuals were sampled across four major river systems (Okavango, Lower Kunene, Lower Shire and Limpopo) and the KwaZulu-Natal region. A multi-marker approach was used to infer phylogeographic and genetic diversity patterns, including new and public mitochondrial data, and a panel of 11 nuclear microsatellites. All individuals belonged to a phylogenetic clade previously associated with the *C*. *niloticus* species, thus suggesting the absence of *C*. *suchus* in southern Africa. The distribution of mitochondrial haplotypes indicated ancestral genetic connectivity across large areas, with loss of diversity along the north-south axis. Genetic variation partitioned the populations primarily into western and eastern regions of southern Africa, and secondarily into the major river systems. Populations were partitioned into five main groups corresponding to the Lower Kunene, the Okavango, the Lower Shire, and the Limpopo rivers, and the KwaZulu-Natal coastal region. All groups show evidence of recent bottlenecks and small effective population sizes. Long-term genetic diversity is likely to be compromised, raising conservation concern. These results emphasize the need for local genetic assessment of wild populations of Nile crocodiles to inform strategies for management of the species in southern Africa.

## Background

Crocodiles (subfamily Crocodylinae), or true crocodiles, are large aquatic, carnivorous reptiles that inhabit tropical and subtropical freshwater lakes, rivers, wetlands, brackish and coastal habitats. Three commonly recognised groups are found across the African continent and Madagascar: the dwarf crocodile (*Osteolaemus tetraspis*) and the slender-snouted crocodile (*Mecistops cataphractus*) are both restricted to central and western Africa [1], whilst Nile crocodiles have a much broader geographic range. Presently, Nile crocodiles can be found from the Nile River in the north and the Senegal River in the west to the Congo Basin, across the Great Lakes in the east, and down to the southernmost limits of the distribution in the Lower Kunene River (Namibia), the Okavango Delta (Botswana), the St. Lucia Wetlands (South Africa), and Madagascar [2].

The total Nile crocodile “meta-population” is estimated to comprise 250,000 to 500,000 individuals, and was classified by the Crocodile Specialist Group as a ‘Low Risk/Least Concern’ single panmictic population in the IUCN Red List of Threatened Species in 1996 for conservation purposes [3]. Due to specific concerns, some countries currently classify their Nile crocodile populations independently. Namibia classifies Nile crocodiles as ‘peripherally endangered’ [4], and in South Africa, the species is considered ‘vulnerable’ [5]. As such, Nile crocodiles benefit from legal protection in many countries.

The classification of Nile crocodiles in different nominal species and subspecies, based on geographically correlated morphological differences, has been long debated [6]. Recently, genetic studies have unfolded a complex and dynamic evolutionary history that resulted in high phylogeographic divergence between populations in different regions, and ultimately in the separation of Nile crocodiles in two distinct non-sister species. Analyses based on mitochondrial DNA (mtDNA) sequences showed that western and central African Nile crocodiles formed a monophyletic group with very low internal divergence, whereas eastern African and Madagascan populations formed another group with slightly higher internal divergence [7]. Based on these findings, the authors proposed a taxonomic revision of Nile crocodiles and the resurrection of *Crocodylus suchus* as the designation for central-western Nile crocodiles. This designation aims to reflect the genetic divergence between *Crocodylus suchus* and the eastern African populations, proposedly designated *Crocodylus niloticus*. Subsequent studies consistently recovered a paraphyletic phylogeny of Nile crocodiles evidencing a monophyletic, ancestral and predominantly ‘*Western clade*’, and a derived predominantly ‘*Eastern clade*’ more recently related to New World species [8–10]. The ‘*Western clade*’ versus ‘*Eastern clade*’ pattern of phylogenetic divergence was also consistent with mitochondrial markers and diagnostic karyotypes [8, 9]. These studies contributed to the recent acceptance of the classification of Nile crocodiles into two species with partially overlapping distributions, *C*. *niloticus* (‘*Eastern clade*’) and *C*. *suchus* (‘*Western clade*’) [11]. The general recognition of the new taxonomic classification is a significant step towards the decryption of the genetic diversity of an important African apex predator, and the derived assumptions from the genetic composition of populations will potentially impact the development of specific conservation strategies.

Although the present study focuses on the population structure of Nile crocodiles in southern Africa, it is relevant to recall the current knowledge of western and central African populations. Nile crocodiles were widespread across the Sahara-Sahel region since the mid-Holocene until the early 20th century, and populations have experienced historical range contractions due to paleogeological events, climatic shifts and anthropogenic pressure [12]. The relict desert-adapted *C*. *suchus* in the Saharan mountains is one such example. A series of census surveys conducted in Mauritania identified fragmented populations, most of which were comprised of less than five individuals. Interestingly, crocodile carcasses were found in dried riverbeds that connect small permanent ‘güeltas’ (rocky pools) and seasonal ‘tâmoûrts’ (wooded floodplains), the most occupied habitats (albeit with limited carrying capacity), thus suggesting the existence of small-scale spatial and genetic connectivity [13]. Genetic analyses performed in the same region confirmed this hypothesis: while mitochondrial lineages revealed absence of genetic structure, compatible with historical panmixia, microsatellite data showed unusually high levels of population structure and genetic differentiation compared to other crocodilian species [14]. The authors explained these results as the outcome of geographic isolation, small population sizes and genetic drift, with limited genetic connectivity occurring mostly within sub-basins, and infrequent overland movements between sub-basins. However, genetic diversity levels in all sub-basins were relatively high and comparable to those reported in other crocodilian species. Similar patterns of extensive genetic differentiation at the drainage basin and landscape (coastal and inland) levels caused by philopatry and restricted gene flow were found throughout western and central Africa [15, 16].

‘*Eastern clade*’ Nile crocodiles (*C*. *niloticus*) have also shown extensive biogeographical genetic sub-structuring associated with major river drainages in eastern Africa (Lake Turkana, and the Great Ruaha, the Zambezi and the Limpopo Rivers) and Madagascar, although only a small number of individuals was sampled at each location (n = 11 to 17) [15]. Again, natural barriers and natal philopatry seemed to be the main contributors to the observed patterns of genetic structure. These results, based on the distribution of microsatellite frequencies and the presence of private alleles, challenged previous assumptions of *C*. *niloticus* uniformity throughout its distribution range. This emerging complex picture of population structure and demographics warrants the necessity for accurate assessments of Nile crocodile populations. This might be especially relevant in the current scenario of climate change and anthropogenic pressure that may have unforeseen impacts on wild animal populations.

The present study provides a ‘finer-scale’ portrait of the current genetic diversity, phylogeography and population structure of Nile crocodiles in southern Africa, with a focus on five important rivers systems/regions: the Lower Kunene River and the Okavango River (Namibia and Botswana, the limit of the distribution in west southern Africa), and the Lower Shire River (Malawi), and the Limpopo and KwaZulu-Natal region in South Africa. Population relationships and contemporary dynamics were inferred using a multilocus approach based on mtDNA control region sequences and nuclear microsatellites.

## Material and methods

### Sample collection and DNA extraction

A total of 149 Nile crocodiles samples were collected from wild populations in three southern African river systems: the Okavango River system (n = 62), the Lower Shire River system (n = 52); the Lower Kunene River system (n = 12); and wild-caught farm-held individuals from two commercial crocodile farms, presumably originating from the Limpopo River or surrounding tributaries (n = 13), and the costal estuarine waterways of KwaZulu Natal (n = 10) in South Africa (S1 Table, S1 Fig). The Okavango River system was subdivided into three sampling sites: the Bwabwata National Park (Namibia, n = 20), the Okavango Delta (Botswana, n = 29), and the Otjiwarongo Crocodile Ranch (Namibia, n = 13), a commercial crocodile farm composed of individuals considered representative of a wild population from the Okavango River. Two sampling sites were targeted in the Lower Shire River system (Malawi) using the Nchalo Sugar Estate (latitude -16.20349; longitude 34.84034) as a landmark: northwards to Kapichira Falls (n = 27), and southwards to the Zambezi Confluence (n = 25).

Blood samples were collected from the ventral caudal tail vein and stored in K2EDTA vacutubes. Tissue samples were collected by the removal of one to two scutes in a unique pattern for future identification of the individual crocodile [17]. All samples were stored at -20°C until DNA extraction. Total DNA was extracted using a CTAB protocol [18], and stored at -20°C. All samples were collected and exported under the appropriate CITES Scientific Authority and the official permits required for each country [Namibia CITES Export No: 0044385; South Africa CITES Import NO: 152009, Research/Collection permit (the Namibian Ministry for Environment and Tourism), permit no. 1881/2014; 2003/2015)]. Ethical clearance for this study was granted by the Stellenbosch University Ethics Committee (Reference no.: SU-ACUD15-00007).

### Mitochondrial DNA sequences

Primers were manually designed based on an alignment of publicly available sequences of the Nile crocodile mtDNA control region (retrieved from S2 Table). A 514-bp fragment was amplified and sequenced for 133 individuals using primers CnP1F (5’-AGTCATCGTAGCTTAACTCACA-3’) and CnP1R (5’-TGTATAACGAGCATTAAATATTTATG-3’). PCR amplifications were performed in a total volume of 10 μl containing 1x KAPA Taq ReadyMix (KAPA Biosystems, Cape Town, SA), 0.8 μM of each primer and 50 μM of DNA, as follows: initial denaturation at 95°C for 5 min, 35 cycles of 95°C for 15 s, 56°C for 30 s and 72°C for 80 s, and a final extension at 72°C for 5 min. Negative controls were included in all DNA extractions and PCR amplifications. Sequencing reactions were performed in the forward direction using the BigDye® Terminator v3.1 sequencing kit (Applied Biosystems, Foster City, CA, USA), following the manufacturer’s recommendations. Electrophoretic separations were performed on an ABI3730xl sequencer (Applied Biosystems) at the Central Analytical Facilities of Stellenbosch University, South Africa. Sequences were aligned using the MUSCLE algorithm implemented in MEGA X [19], after manual correction of ambiguities in base calling.

### Mitochondrial diversity, population structure and phylogenetic reconstruction

Standard diversity measures (number of haplotypes, haplotype diversity, nucleotide diversity, and average number of pairwise nucleotide differences) were estimated for each population using Arlequin software v3.5 [20]. A median-joining network was constructed to illustrate evolutionary relationships among haplotypes using Network v4.6.3, under default settings [21]. Publicly available sequences and information on their sample collection sites were retrieved from Dryad Digital Repository (http://datadryad.org/resource/doi:10.5061/dryad.s1m9h/3) [15] (S2 Table).

### Microsatellite selection, multiplexing and genotyping

Twelve nuclear microsatellite loci previously described for *Crocodylus porosus* and *Crocodylus johnstoni*, and tested in *C*. *niloticus*, were selected for PCR amplification based on number of alleles (A_n_ > 6) and observed heterozygosity (H_o_ > 0.300) [22, 23]. Six samples (two from each river system) were used for initial singleplex gradient PCR tests to assess optimal annealing temperatures (T_a_) and marker polymorphism. Marker *CpP305* [24] was included in the preliminary tests, but this locus was subsequently excluded due to ambiguity in allele calling. Three multiplex PCRs were designed based on T_a_, expected allele range, and fluorescent labels. Due to T_a_ and fluorescent label constrictions, marker *C391* was amplified independently, but PCR products were pooled with those of Multiplex 2 (S3 Table). Multiplex PCR amplifications were performed in a 10 μl total volume containing 1x KAPA2G Fast Multiplex PCR Kit (KAPA Biosystems), 0.8 μM of each primer and 50 μM DNA, as follows: initial denaturation at 95°C for 3 min, 35 cycles of 95°C for 15 s, T_a_ for 30 s, 72°C for 50 s, and a final extension at 72°C for 80 s. Negative controls were included in all amplifications. PCR products were separated on an ABI3730xl Genetic Analyser^™^ (Applied Biosystems) with GeneScan^™^ 600 LIZ® (Applied Biosystems) as the internal size standard. Genotypes were scored using GeneMapper® v4.1 (Applied Biosystems). The presence of null alleles and scoring errors due to stuttering was tested for each locus using Micro-Checker v2.2.3 [25] (1,000 replicates with 95% confidence intervals).

### Nuclear diversity measures and population structure

Departures from Hardy-Weinberg equilibrium (HWE) (exact probability test, 500 batches, 10,000 iterations), number of alleles (A_n_), expected (H_e_) and observed heterozygosity (H_o_), and the fixation index (F_is_) were calculated using GenAlEx v6.5 [26]. Rarefied allelic richness (R_s_) was estimated using HP-RARE v1.1 [27]. Polymorphic information content (PIC) was calculated using Microsatellite Tools v3.1 (http://animalgenomics.ucd.ie/sdepark/ms-toolkit/index.ph). Pairwise F_st_ and a locus-by-locus hierarchical AMOVA (significance testing at the 1% nominal level, using 1,000 permutations) were calculated using GenAlEx v6.5. For the AMOVA, the sampling populations were grouped into five regions based on river system or geographic origin, as follows: Okavango (Namibia and Botswana), Lower Kunene (Namibia), Lower Shire (Malawi), Limpopo (South Africa), and KwaZulu-Natal (South Africa). Principal coordinate analysis (PCoA) based on the genetic distance with variance standardisation was also performed using GenAlEx v6.5. Ancestral population structure was inferred using STRUCTURE v2.3.4 [28]. An initial analysis was performed on the total dataset for *K*-values between 1 and 16 (twice the number of sampling populations; 10 replicates for each *K*; 50,000 steps of burn-in period followed by 500,000 steps of MCMC), under the admixture model with independent allele frequencies, without *a priori* population information. The most likely number of population clusters (*K*) was estimated using a variety of statistics, including LnPr(X|*K*), Δ*K* [29], as well as four newer statistics (MedMed *K*, MedMean *K*, MaxMed *K*, MaxMean *K*) based on the Puechmaille method [30], using StructureSelector [31]. Individual assignment to genetic clusters were visualised using CLUMPAK [32]. Mean relatedness [33] for each population cluster (as estimated by STRUCTURE) was also calculated with 95% confidence about the mean tested by bootstrap resampling (1,000 iterations), and significant differences between the population clusters tested through permutation (95% CI, 1,000 iterations) in GenAlEx.

### Effective population size and bottleneck estimates

Contemporary effective population sizes (N_e_) were estimated using the linkage disequilibrium (LD) method, assuming a random mating model for the populations inferred in the STRUCTURE analysis, as implemented in NeEstimator v2.01 [34] (significance testing: 95% CI with 1,000 permutations). Testing for recent bottlenecks or radial expansion was performed using the Wilcoxon signed rank test for significant deviation due to heterozygosity excess or deficiency under the Infinite Allele Model (IAM) and the Stepwise Mutation Model (SMM) implemented in Bottleneck v1.2.02 [35]. Bottleneck analyses were performed using 1,000 replications at the 5% nominal level, and mode shifts in allele frequency distributions were also inspected.

### Estimation of directionality and magnitude of migration in populations displaying genetic connectivity

The manner in which wild populations interact in a river system was determined using the program Migrate-N [36]. The migration patterns in the Okavango (OR) and Shire (SH) were modelled to investigate behavioural patterns in the species. In general, two models were tested for both river systems: 1) a full model with distinct populations, with migration to and from each population; and 2) a model assuming complete panmixia between all populations. For the OR, two additional models were tested: 3) a model assuming migration from Botswana to Namibia; and 4) a model assuming migration from Namibia to Botswana. Similarly for the SH, asymmetric gene flow between northern and southern regions of SH were assessed, assuming: 3) migration from the northern Shire to the southern Shire; and conversely 4) migration from the southern Shire to the northern Shire. The mutation scaled effective population size Θ = 4N_e_μ, where N_e_ is the effective population size and μ is the mutation rate per generation per locus, and mutation- scaled migration rates M = m/μ, where m is the immigration rate per generation, among regions, was also calculated in Migrate-N. A Brownian process was used to model microsatellite mutations, and ran using random genealogy and values of the parameters Θ and M produced by F_ST_ calculation as a starting condition. Bayesian search strategy was conducted using the following parameters: an MCMC search of 5 × 10^5^ burn-in steps followed by 5 × 10^6^ steps with parameters recorded every 1,000 steps. A static heating scheme with four different temperatures (1.0, 1.5, 3.0 and 1 × 10^6^) was employed, where acceptance–rejection swaps were proposed at every step.

## Results

### Mitochondrial phylogeography and genetic diversity

**Fig 1:**
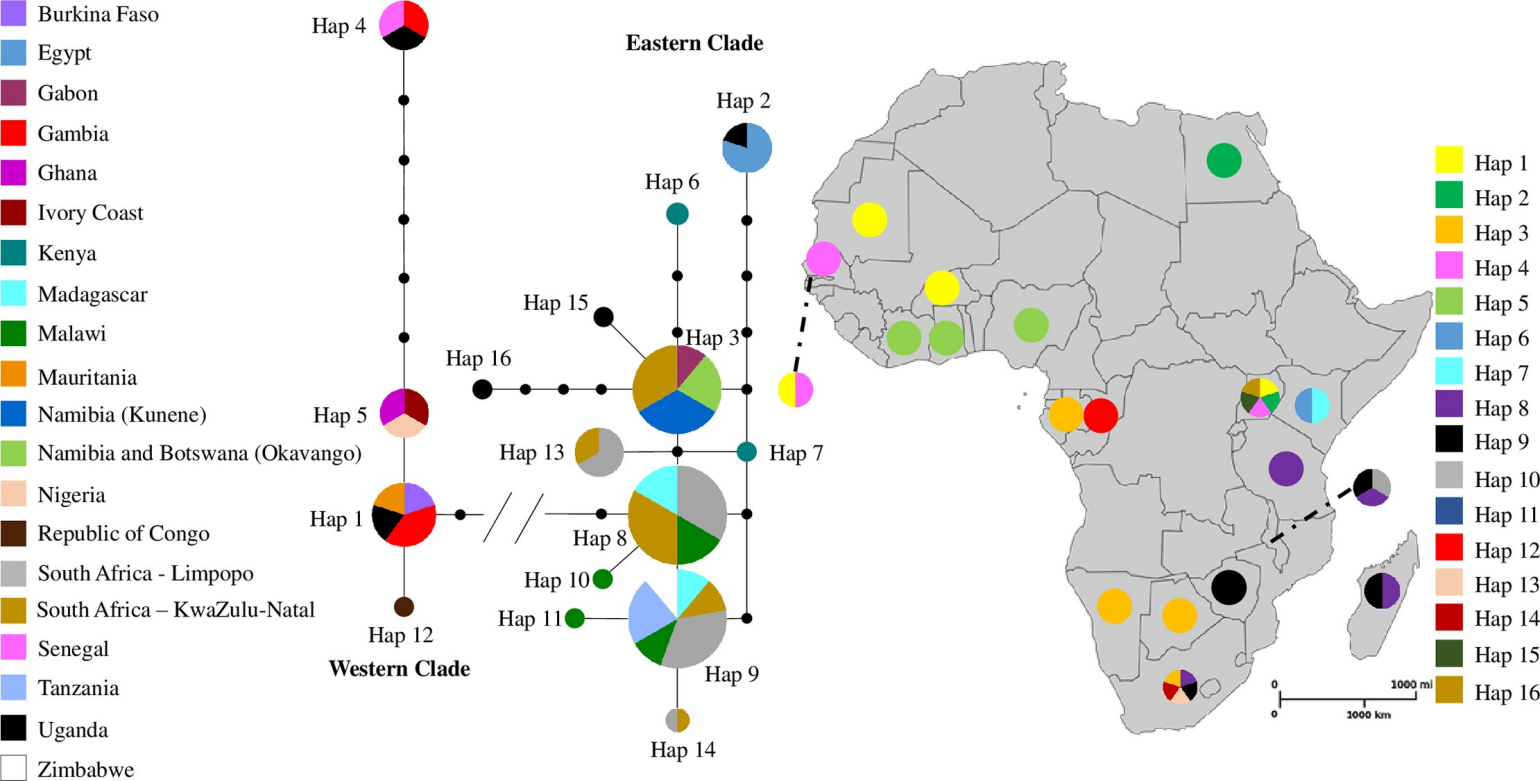
Median-joining network of Nile crocodile (*C*. *niloticus* and *C*. *suchus*) and geographic distribution of haplotypes. Based on a 516 bp fragment of the mtDNA control region, 16 haplotypes in continental Africa and Madagascar using new and previously published sequences were observed. The size of the circles is proportional to the frequency of the haplotype; lines represent a single substitution step; black dots represent hypothetical unobserved haplotypes; // represents 16 mutational steps.

The median-joining network (Fig 1) showed the following: all individuals from Namibia, Botswana, Malawi and South Africa clustered in the previously reported ‘*Eastern clade*’ [7, 15], therefore strongly suggesting the exclusive presence of *C*. *niloticus* in the regions surveyed in this work. Only five haplotypes were found amongst the Nile crocodiles from the southern African river systems. These haplotypes were defined by seven variable sites, all of which consisted of transitions. Strikingly, all individuals from the Lower Kunene River system (n = 12) and the Okavango River system (n = 62) had the same haplotype (Hap 3), that was also shared with Gabon and the KwaZulu-Natal region of South Africa (Fig 1). In contrast, four haplotypes were found in the Lower Shire River (n = 52), two of which (Hap 10, n = 2 and Hap 11, n = 2, in the total dataset) were unique, and are reported here for the first time. Of the other two haplotypes, one was shared with Madagascar and South Africa, and had been previously reported in Malawi (Hap 8, n = 12 in the total dataset). The other haplotype (Hap 9, n = 10 in the total dataset) was shared with Tanzania, Zimbabwe, South Africa, and Madagascar [15]. The unique haplotypes were one mutational step derived from shared haplotypes also found in Malawi (Fig 1), and no haplotypes were shared between the Lower Shire and the Lower Kunene/Okavango Rivers (Botswana/Namibia). Therefore, the eastern southern African Nile crocodiles (Lower Shire, Limpopo and KwaZulu-Natal) had high haplotype diversity, with the highest haplotype diversity overall found in the KwaZulu-Natal individuals (h = 0.861 ± 0.087; π = 0.006 ± 0.001) (S4 Table).

### Microsatellite-based genetic diversity and genetic structure estimates

The 11 markers amplified in more than 95% of the samples, with the exception of *CpP307* and *Cj18* which failed to amplify in 19% and 24% of the samples, respectively, most probably due to intra-specific sequence polymorphisms. There was limited evidence for genotypic artefacts, such as null alleles and stuttering (S5 Table). Most loci did not depart from HWE expectations, with the exception of *CpP307* in the Lower Shire River (North and South) samples, and *CpP1409* in the Okavango Delta Botswana samples (S5 Table). A total of 122 alleles was found across all loci, with the number of alleles per locus varying between four (*C391 and CpP309*) and 29 (*CpP1409*). Most loci showed moderate values of H_e_ (≥ 0.55) and H_o_ was slightly lower than H_e_ for most loci, except for the Okavango Delta Namibia and South African samples, when considering each river system independently (S5 Table).

The Lower Shire River system had the highest diversity (H_e_ = 0.67; H_o_ = 0.62; R_s_ = 4.29, averaged across the north and south samples), followed by the South African groups (H_e_ = 0.64, H_o_ = 0.55, and R_s_ = 3.78, averaged across Limpopo and KwaZulu-Natal), the Okavango River system (H_e_ = 0.61, H_o_ = 0.59 and R_s_ = 3.65, averaged across the three groups), and finally the Lower Kunene River system (H_e_ = 0.58, H_o_ = 0.50, and R_s_ = 3.31). Fixation indices (F_is_) indicated a slight excess of homozygotes in the Lower Kunene River (F_is_ = 0.149) and South Africa as a whole (F_is_ = 0.182), as well as the Lower Shire River (North, F_is_ = 0.098; and South, F_is_ = 0.071), and the KwaZulu-Natal region (F_is_ = 0.348). In contrast, the fixation indices were low for Okavango River system (Okavango Delta Botswana, F_is_ = 0.011; Bwabwata National Park, F_is_ = -0.009; and Okavango Delta Namibia, F_is_ = 0.043). However, departures from HWE were non-significant when considering each sampled population separately. On the contrary, over all cohorts, as one, HWE departures were found to be significant due to heterozygous deficiency (Table 1).

**Table 1:** Summary of genetic diversity measures and test for Hardy-Weinberg Equilibrium (HWE) in southern African Nile crocodile populations. OR-BNP-Nam: Okavango River system—Bwabwata National Park–Namibia, OR-OD-Bot: Okavango River system—Okavango Delta—Botswana, OR-OCR-Nam—Okavango Crocodile Ranch—Okavango River—Namibia, LK-Nam–Lower Kunene River—Namibia, LS-N-Mal–Lower Shire River (North)—Malawi, LS-S-Mal–Lower Shire River (South)—Malawi, and Limpo-SAf–Limpopo River—South Africa, KZN-SAf–KwaZulu-Natal—South Africa. N—number of individuals, A_n_—number of alleles, R_s_—mean allelic richness, H_e_—expected heterozygosity, H_o_—observed heterozygosity, and F_IS_—mean fixation index. Values were estimated based on genotypes determined using 11 nuclear microsatellite markers and averaged across all loci.

Population	N	A_n_	R_s_	H_e_	H_o_	F_is_	HWE (*P*-value)
OR-BNP-Nam	20	4.6	3.64	0.600	0.549	-0.009	0.616
OR-OD-Bot	29	6.0	3.73	0.624	0.613	0.011	0.486
OR-OCR-Nam	13	5.0	3.58	0.606	0.619	0.043	0.537
**Okavango River (TOTAL)**	**62**	**5.2**	**3.65**	**0.610**	**0.594**	**0.001**	**0.554**
**Lower Kunene River (LK-Nam)**	**12**	**4.2**	**3.31**	**0.583**	**0.495**	**0.149**	**0.587**
LS-N-Mal	27	6.9	4.29	0.664	0.617	0.098	0.337
LS-S-Mal	25	6.9	4.28	0.684	0.625	0.071	0.367
**Lower Shire River (TOTAL)**	**52**	**6.9**	**4.29**	**0.674**	**0.621**	**0.085**	**0.352**
Limpo-SAf	13	4.7	3.73	0.634	0.717	-0.104	0.273
KZN-SAf	10	4.4	3.82	0.639	0.390	0.348	0.249
**South Africa (TOTAL)**	**23**	**4.6**	**3.78**	**0.637**	**0.554**	**0.182**	**0.261**
**Total dataset**	**149**	**11.7**	**3.76**	**0.723**	**0.594**	**0.144**	**0.002**

**Fig 2:**
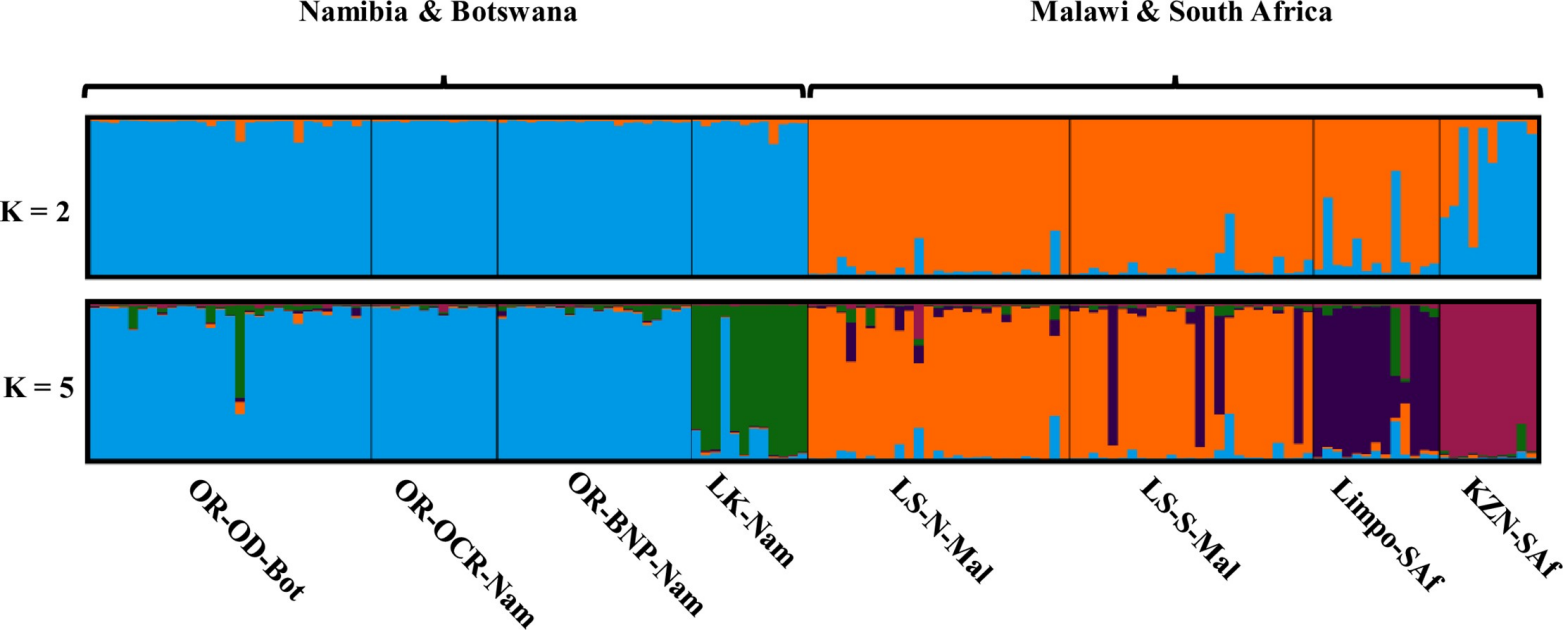
STRUCTURE bar plot showing the distribution of Nile crocodile populations. The most likely number of population clusters, *K* = 2 (based on Δ*K*, representing the upper hierarchical level) and *K* = 5 (based on all other estimates, giving a more “localised” regional evaluation of population structure). [OR-BNP-Nam: Okavango River system—Bwabwata National Park–Namibia, OR-OD-Bot: Okavango River system—Okavango Delta—Botswana, OR-OCR-Nam—Okavango Crocodile Ranch—Okavango River—Namibia, LK-Nam–Lower Kunene River—Namibia, LS-N-Mal–Lower Shire River (North)—Malawi, LS-S-Mal–Lower Shire River (South)—Malawi, Limpo-SAf–Limpopo River—South Africa, KZN-SAf–KwaZulu-Natal—South Africa].

**Fig 3:**
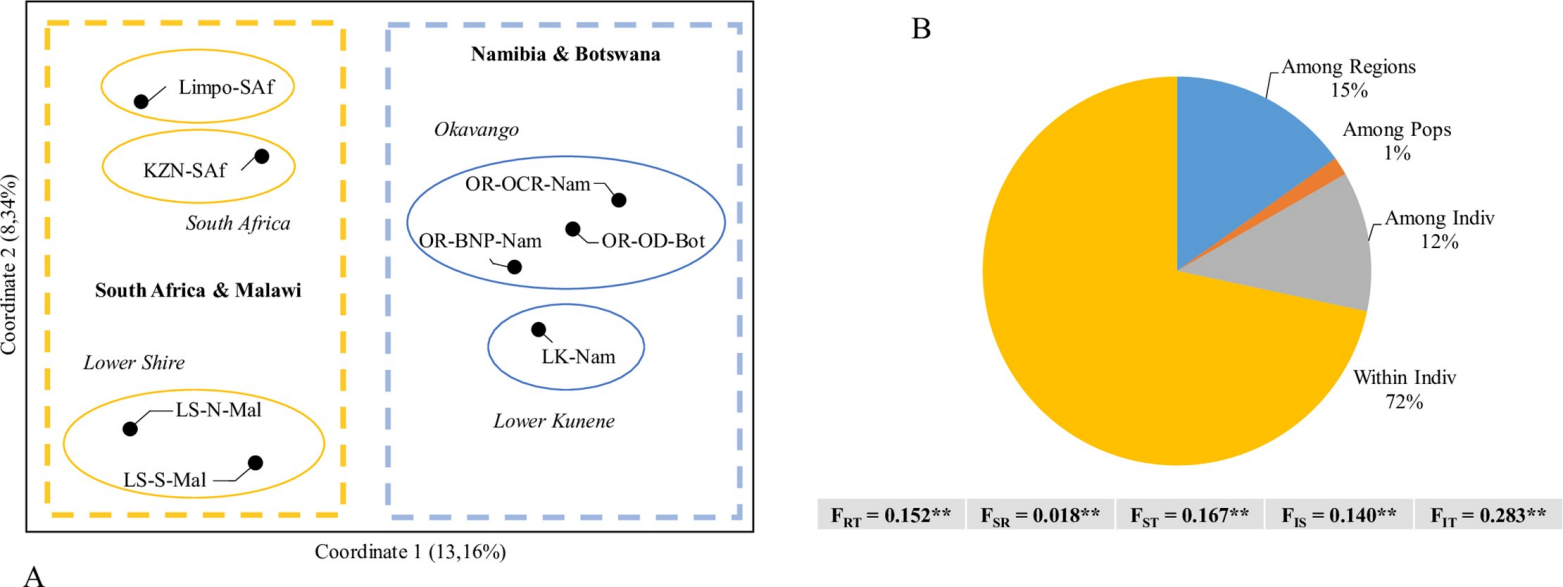
Multivariate analyses of population structure of the Nile crocodile in southern Africa. (A) Principal Coordinate Analysis (PCoA) showing the upper hierarchical population clusters, east and west (primarily differentiated by the first coordinate) as well as the ‘secondary’ population clusters representing each of the regional river systems (Okavango River, Lower Kunene River, Lower Shire River, Limpopo and KwaZulu-Natal regions). [OR-BNP-Nam: Okavango River system—Bwabwata National Park–Namibia, OR-OD-Bot: Okavango River system—Okavango Delta—Botswana, OR-OCR-Nam—Okavango Crocodile Ranch—Okavango River—Namibia, LK-Nam–Lower Kunene River—Namibia, LS-N-Mal–Lower Shire River (North)—Malawi, LS-S-Mal–Lower Shire River (South)—Malawi, and Limpo-SAf–Limpopo River—South Africa, KZN-SAf–KwaZulu-Natal—South Africa.] (B) Molecular Analysis of Variance (AMOVA) showing significant population differentiation between river systems, but also amongst ‘sub-populations’ within river systems, although most of the genetic variation can be ascribed to within individual variation (** statistically significant at the 1% nominal level).

The number of population clusters based on Δ*K* was estimated at two, broadly representing western (Namibia and Botswana) and eastern (Malawi and South Africa) clusters in southern Africa. Interestingly, the KwaZulu-Natal population did show significant overlap with the western cluster, with K = 2. All other Bayesian statistics estimated the number of populations as five, with each of the river systems representing individual population clusters (Lower Kunene River, Okavango River system, Lower Shire River, Limpopo, and KwaZulu-Natal) (Figs 2 and 3; S2 Fig). Similarly, the PCoA (Fig 3A) showed primary separation of populations into eastern (Malawi and South Africa) and western (Namibia and Botswana) clusters along the first coordinate that explains 13.16% of the variation. The second coordinate (explaining 8.34% of variation) partitioned the sampling populations further into clusters associated with individual river systems. This partitioning was supported by the AMOVA that ascribed a significant percentage of genetic variation (15%) to differences amongst the regions (individual river systems, F_RT_ = 0.152, *P* < 0.01). Subtle population differentiation amongst the sub-populations within each region was also supported (F_SR_ = 0.018, *P* < 0.01) (Fig 3B). The broad assessment of population structure was further reflected more directly in the pairwise F_ST_ comparisons, with the highest genetic distances (F_ST_ ≈ 0.2, *P* < 0.05) found between Limpopo-KwaZulu-Natal, and Lower Kunene River-Limpopo (Table 2). Genetic distances between the Okavango River and the Lower Kunene River were intermediate in the context of this dataset (F_ST_ = 0.090–0.116, *P* < 0.05). The two Lower Shire River cohorts (North and South) were the least differentiated samples (F_ST_ = 0.013, *P* < 0.05), as well as the two wild populations from the Okavango River system (Bwabwata National Park-Okavango Delta, F_ST_ = 0.029, *P* < 0.05). This low genetic differentiation was supported by approximately equal migration rates between sampling sites in the Okavango (S6 Table). However, migration in the Lower Shire River appeared to occur predominantly unidirectional, from the northern to the southern parts of the river (S7 Table).

**Table 2:** Genetic divergence amongst Nile crocodile populations in southern Africa: Lower Kunene River (LK-Nam), Okavango River System (Okavango Delta—OR-OD-Bot and Okavango River—OR-OCR-Nam), North and South Lower Shire River (LS-N-Mal and LS-S-Mal), and South Africa's Limpopo (Limpo-SAf) and KwaZulu-Natal (KZN-SAf) regions. Pairwise F_ST_ values calculated using microsatellite genotypes are represented below the diagonal line.

Population	LK-Nam	OR-BNP-Nam	OR-OD-Bot	OR-OCR-Nam	LS-N-Mal	LS-S-Mal	Limpo-SAf	KZN-SAf
**LK-Nam**	-							
**OR-BNP-Nam**	0.097*	-						
**OR-OD-Bot**	0.090*	0.029*	-					
**OR-OCR-Nam**	0.116*	0.019*	0.012	-				
**LS-N-Mal**	0.188*	0.150*	0.161*	0.176*	-			
**LS-S-Mal**	0.168*	0.128*	0.140*	0.157*	0.013*	-		
**Limpo-SAf**	0.265*	0.207*	0.195*	0.221*	0.130*	0.169*	-	
**KZN-SAf**	0.259*	0.234*	0.223*	0.210*	0.212*	0.203*	0.266*	-

*Significant differentiation (*P* < 0.05)

### Effective population size, growth and contraction, and mean relatedness

Point estimates for effective population size ranged from 115.9 to infinite amongst the various clusters (Table 3). Nonetheless, the lower confidence bound for the Lower Kunene River, Limpopo and KwaZulu-Natal sampling populations were below the absolute critical 50 value. The upper bound estimate for the Okavango River was lower than 500, normally considered as a genetically healthy and sustainable population. The Lower Shire River point and lower bound estimate was also lower than 500, while the upper bound estimate was at 868.1.

**Table 3:** Estimates of contemporary N_e_ of four geographic population clusters of Nile crocodiles based on the linkage disequilibrium (LD) method; and the heterozygosity excess (H_e_) test as an indication of population expansion and contraction based on heterozygosity excess/deficiency under the infinite alleles (IAM) and stepwise mutation (SMM) models (Wilcoxon-sign rank test, *P*-value), and allele frequency distribution mode shifts.

Population Group	N_e_ (95% CI)	Evidence for Population Expansion/Contraction				
		Wilcoxon-sign rank test P-value (IAM)		Wilcoxon-sign rank test P-value (SMM)		Allele Frequency Mode Shift (Y/N)
		H_e_ def	H_e_ exc	H_e_ def	H_e_ exc	
Kunene River	∞ (19.8 - ∞)	1.000	0.000	0.999	0.001	Y
Okavango River	115.9 (76.6–213.9)	1.000	0.000	0.998	0.003	Y
Lower Shire River	199.0 (107.5–868.1)	1.000	0.000	0.992	0.011	N
Limpopo, South Africa	∞ (25.5 - ∞)	1.000	0.001	0.999	0.002	Y
KwaZulu-Natal, South Africa	∞ (17.0 - ∞)	0.793	0.232	0.139	0.880	Y

Significant heterozygous excess (*P* < 0.01), usually indicating a recent population bottleneck, was found in all population clusters under both the IAM and the SMM, except for KwaZulu-Natal (Table 3). Evidence for such bottlenecks was also supported by a mode shift in allele frequency distributions for all population clusters, except for the Lower Shire River (Table 3). Relatedness amongst individuals within each population cluster was significantly greater than zero, with the exception of the Okavango River. The relatedness coefficients ranged from -0.014 (Okavango River) to 0.405 (KwaZulu-Natal). The degree of relatedness was also significantly different amongst the population clusters (S3 Fig).

## Discussion

This study reports the survey of genetic diversity and phylogeography of Nile crocodiles from five different southern African regions: the Lower Kunene River (Namibia), the Okavango River system (Namibia and Botswana), the Lower Shire River (Malawi), and Limpopo and KwaZulu-Natal (South Africa). This work aimed to contribute to the general understanding of ancestral and recent population history of Nile crocodiles by exploring novel genetic data at the fringes of the geographic distribution of the species in southern Africa.

### *Crocodylus suchus* is seemingly absent from southern Africa

Previous analyses of Nile crocodile mitochondrial haplotypes and their geographic distribution support the existence of two diverged evolutionary branches. One of the branches was identified as the ‘*Western clade*’ and currently represents the ‘rediscovered’ species *Crocodylus suchus*, and the other branch was identified as the ‘*Eastern clade*’ and represents the *Crocodylus niloticus* species [15, 37].

The range of the distribution of *C*. *suchus* is presently defined as western and central Africa, although the species also existed in northern Africa (the Nile River) until at least 100 years ago [15, 16]. The broad-scale pattern of the geographic distribution of ‘*Eastern clade*’ and ‘*Western clade*’ lineages suggested that *C*. *suchus* was absent from southern Africa, although the precise limits of the distribution of the species were uncertain [15]. *Crocodylus suchus* was found in the Republic of the Congo (Likouala Aux Herbes), and the species is sympatric with *C*. *niloticus* in Uganda (Kidepo Valley), on the border with South Sudan and Kenya [15, 16]. *Crocodylus suchus* was also found in the Congo River (Kinshasa, Democratic Republic of the Congo), suggesting that it may be sympatric with *C*. *niloticus* throughout the Congo Basin, and possibly further to the south into Angola. All individuals sampled in this study in west, southern Africa (Lower Kunene River and Okavango River) belonged to the ‘*Eastern clade*’, and therefore can be identified as *C*. *niloticus*. As the Congo Basin remains poorly assessed, and the large area southwards (*e*.*g*. Angola) has not yet been surveyed, the precise limit of the geographic range of *C*. *suchus* is presently not defined. However, our study confirms that *C*. *suchus* and *C*. *niloticus* are not sympatric in the southernmost fringe of its distribution in west southern Africa. As for east southern Africa, we did not find *C*. *suchus* in the Lower Shire River (southern Malawi) or South Africa, although both species were found further to the North of the Great Lakes, in Uganda [15]. The presence of *C*. *suchus* in the Great Lakes is still poorly assessed, as only a few individuals were sampled across the area [15, 16]. Nevertheless, the present survey of the Lower Shire River strongly suggests that *C*. *suchus* is absent from Lake Malawi, the southernmost limit of the Great Lakes region, as well as from South Africa.

### *Crocodylus niloticus* shows ancestral genetic continuity across central and western Africa

All individuals sampled in Botswana and Namibia shared Hap 3, a haplotype also found in Gabon [15], with two Ugandan haplotypes (Hap 15 and 16) diverging from this central haplotype. This suggests genetic continuity along west southern Africa (North-South, between Botswana/Namibia and Gabon), and across central Africa (East-West, between Gabon and Uganda) (Fig 1). The geology and hydrology of the region might explain the ancestral genetic continuity of *C*. *niloticus* across central Africa. The Congo Basin consists of closely intertwined river systems that allowed for gene flow from Uganda (Lake Edward and Lake Albert) to the further reaches of the basin in the west. In contrast, the geographic elevation of the eastern region of the Congo Basin resulted in river systems that flowed in an easterly direction to flow westward, towards Lake Victoria, forming the current Victoria-Edward region, at around 500,000 years ago [38]. This may also explain the finding of exclusive haplotypes in the Tana River (Hap 6 and Hap 7, Kenya), which has its source in the Aberdare Mountains and flows eastwards to the Indian Ocean, because of its long-term isolation from the region known as the Kenya Dome.

Genetic continuity along the North-South axis is also evident from the sharing of Hap 3 between Bostwana/Namibia and Gabon. The presence of ‘*Eastern clade*’ haplotypes in western Africa was hypothesised to be most likely the result of a “northward dispersal of *C*. *niloticus* from coastal Angola and the Kunene River” [15]. Crocodiles are known to be able to travel long distances in salt water, and to make use of ocean currents [39]. The sharing of haplotypes between eastern Africa and Madagascar (Hap 8 and 9) is a good example of the sea faring capacity of *C*. *niloticus*. Nile crocodiles have also been observed several kilometres off the shore of Gabon (Matthew Shirley, pers. comm.). However, ocean currents are warmer closer to the equator, and crocodilians are ectothermic, *i*.*e*. rely on the environment for body temperature control, with cold temperatures limiting their movements. The seawater temperature along the west coast of Africa between Gabon and the Kunene River is defined by sea currents: the warm Angola Current flows from the Gulf of Guinea to southern Angola, where it meets the cold northwards-flowing Benguela Current forming the Angola-Benguela Frontal Zone. The cold seawater temperatures in this area probably constitute a barrier to the migration of crocodiles northward of the Kunene River. Hence, the sharing of Hap 3 between Gabon and Botswana/Namibia more likely resulted from a dispersal of *C*. *niloticus* throughout the Congo Basin and southwards towards the Zambezi River system. The Kasai River, a southern boundary river system of the Congo Basin, is a good candidate for a connector between the Congo Basin and the Zambezi River, as the Kasai was pirated by the Upper Zambezi River system in recent geological times [40]. The southern flow of the Upper Zambezi River and the closely connected systems of the Zambezi region in north eastern Namibia likely allowed crocodiles to disperse into neighbouring river systems, such as the Kwando and the Okavango. Therefore, it seems more likely that the sharing of Hap 3 between Gabon and Botswana/Namibia is the result of a land dispersal from Uganda across the Congo Basin towards the Kunene and the Okavango river systems, rather than the result of an oceanic dispersal.

The sharing of Hap 3 by all individuals sampled in Namibia and Botswana (n = 65) evidences a common ancestral origin that can be explained by ancient geographic features. The Kunene River and the Okavango River are presently separated by an arid region, the Cuvelai-Etosha Basin. The Upper Kunene River was a major tributary of this basin, which harboured many water-dependent species [41]. The presence of Kobus leche (red lechwe), Clariidae fish fossils [42], and Nile crocodile fossils [43] suggests that the Kunene River basin and the Okavango River basin shared the same species [44]. With the inland erosion of the Lower Kunene River, a piracy event occurred in the Calueque area (southern Angola), resulting in the deflection of the Upper Kunene River towards the Atlantic Ocean in the Late Pleistocene period, and the slow aridification of the Cuvelai-Etosha Basin [41, 45]. However, a great paleo-lake persisted until 35,000 years ago, at which point the aridification process of the basin was finally complete [41]. This water body would have allowed crocodiles to exist in the region as a single panmictic population for a prolonged period. The presence of a single haplotype in the Lower Kunene River and the Okavango River is also in agreement with the fact that the two river systems harbour the most south-western *C*. *niloticus* populations of the African continent. Geographic dispersal from a centre of origin often represents a founding event or a series of founding events that may result in loss of diversity and the fixation of haplotypes in populations [46].

Interestingly, several crocodiles sampled from KwaZulu-Natal, South Africa had Hap 3, suggesting shared ancestry with crocodiles from Botswana and Namibia. Several palaeo-environmental studies have identified signatures of progressive aridification in southern Africa, which led to the conversion of swamplands to the current semi-dessert Karoo and Kalahari regions [47]. Thus, the previously suitable habitats may have facilitated the movement of *C*. *niloticus* between and within Namibia, Botswana and South Africa, as far south as modern KwaZulu-Natal [47, 48].

### *Crocodylus niloticus* also shows ancestral genetic continuity along eastern Africa and Madagascar

A second group of haplotypes evidences long-range genetic continuity throughout the region that stretches from Tanzania to South Africa, including Madagascar. This area shares a distinct sub-cluster of four ‘*Eastern clade*’ haplotypes found in Lake Rukwa and the Rufiji River (Tanzania), Lake Kariba (Zimbabwe), the Lower Shire River and Lake Malawi (Malawi), Lake St. Lucia, Limpopo and KwaZulu-Natal farms (South Africa), and Madagascar (Fig 1). The two new private haplotypes found in the Lower Shire River (Hap 10 and Hap 11), being one-mutational step derived from ‘central haplotypes’, further contribute to the unfolding of this distinct sub-cluster. The geographic distribution of the sub-cluster is compatible with a separation of ancestral populations during the formation of the east African Rift Valley.

The sharing of Hap 9 between Lake Malawi and its neighbouring river systems supports genetic continuity amongst eastern African regions. In the past, Lake Rukwa was a much larger water body that could have served as a connection for aquatic animals between Lake Tanganyika and Lake Malawi [49]. In the future, it will be interesting to survey Lake Tanganyika for the presence of Hap 3 and Hap 9 in the southern part of the lake, as geological evidence to support connectivity has been considered insufficient [50]. Lake Malawi is presently connected to the Shire River, its only outlet flowing south into the Zambezi River. Kapichira Falls separate the Shire River system in the Upper Shire and Lower Shire Rivers, and constitutes a barrier for the northward migration of fish species from the Zambezi River into Lake Malawi [51]. Thus, the presence of Hap 9 in Lake Kariba most likely reflects a westwards dispersal of *C*. *niloticus* from Lake Malawi into the Zambezi River system.

The striking exclusivity of this group of related haplotypes (Hap 8, Hap 9, Hap 10 and Hap 11) in the region, as well as in Madagascar, evidences the common origin and dispersal of a specific ancestral population of Nile crocodiles across the eastern part of southern Africa, most probably down to the southernmost limit of the distribution of the species. Haplotype 9 and Hap 8 (Fig 1), were found in the Limpopo and KwaZulu-Natal, which points to shared ancestry amongst all South African *C*. *niloticus* populations. It is possible that ancestral Nile crocodiles have moved with the Limpopo River into the Indian Ocean, and followed the warm oceanic current along the eastern coast of South Africa to colonise the KwaZulu-Natal region, as reported previously [39]. The increased number of haplotypes found in South African could be explained by the sourcing of wild crocodile from a variety of locations to establish the farmed populations (Fig 1; S4 Table).

### Uganda as a hotspot for genetic Nile crocodile diversity

The overall analysis of haplotype distribution suggests that Uganda is an ancestral and contemporary hub for Nile crocodile diversity (Fig 1). Both *C*. *suchus* and *C*. *niloticus* have been found in the region, evidencing the connections to the western limit of the Congo Basin and the Nile Basin, where *C*. *suchus* was present until recent times [8]. The intra-specific haplotype diversity in Uganda was exceptionally high, with two *C*. *suchus* haplotypes (Hap 1 and Hap 4, shared with western Africa), as well as three diverged *C*. *niloticus* haplotypes (Hap 2, Hap 15 and Hap 16). This suggests that the Great Lakes region may presently harbour the oldest crocodile populations in Africa; however, vast areas of the continent have never been surveyed and new data may originate a competing hypothesis. Hap 2 was also found in the Nile River, thus further supporting genetic continuity between the Great Lakes and the Nile Basin [8]. Haplochromine cichlids in Lake Victoria have also shown a pattern of closer relationship with species found in the Congo River and Nile River basins than with eastern African species [52].

Kenya (Tana River, n = 3) has a distinct profile with the presence of two exclusive haplotypes. This observation is, however, compatible with the region having become isolated from Uganda and Tanzania by mountain ranges that were most likely formed during the same period as the east African Rift Valley.

### Contemporary population dynamics of southern African populations based on nuclear microsatellite markers

The microsatellite analyses consistently showed a distinction between western (Namibia and Botswana) and eastern (Malawi and South Africa) *C*. *niloticus* populations, in accordance with the mtDNA analysis. This distinction represents the upper hierarchical population structure detected with STRUCTURE using the Δ*K* method (Fig 2 and S2 Fig) [31]. The genetic differentiation is most probably the result of ancestral separation of populations into broad geographic regions, in congruence with the trend for an east-west divide supported by the phylogeographic structure of Nile crocodiles in southern Africa. A previous study, based on microsatellite analysis, showed clear signatures of genetic structure within major river systems in east Africa and Madagascar [15], and up to eight genetic clusters for *C*. *suchus* were identified in western- and central Africa [16]. However, the current study only found marginal evidence for population stratification within major river systems or geographical regions in southern Africa, as the five ‘main’ population genetic clusters represented the five river systems and geographic regions. This high degree of genetic homogeneity across sampling populations within geographical regions, both at the mtDNA and at the nuclear marker levels, can be at least partially explained by the exceptionally slow mutation rate of crocodilian genomes, hypothesised to be the result of consistently long generation times over the course of the evolution of the group [52]. Low levels of mtDNA structuring have been reported for other long-lived species [53], including the American alligator (*Alligator mississippiensis*), and may be partially explained by low metabolic rates resulting in low mutation rates after ancient bottlenecks [54]. However, evidence for contemporary population structure for other African crocodile populations [15, 16], suggests that gene flow within southern African river systems are high, but that animals rarely move between river systems. Preliminary estimates of migration rates, although with some uncertainty, appear to support gene flow within river systems, as migration appeared to occur between all locations in the Okavango River (S6 Table), as well as in a north to south direction in the Lower Shire (S7 Table). Interestingly, the South African populations, Limpopo and KwaZulu-Natal, clustered individually as distinct populations (K = 5), and the KwaZulu-Natal population seemed to share ancestry with the *Western clade* (K = 2) (Fig 2). This is in accordance with KwaZulu-Natal sharing a haplotype with several western populations, (Hap 3, Fig 1), further supporting an ancestral connection between these regions during the Palaeozoic Era, and the subsequent isolation of the KwaZulu-Natal population.

Whereas amongst population variance is low, within population diversity is a major contributor to genetic variation (as evidenced by AMOVA, Fig 3B). High levels of exploitation have led to drastic population declines during the mid-20th century [12, 55]. This would suggest that genetic diversity was negatively impacted in many crocodile populations. However, Hekkala et al. [15] reported moderate levels of diversity that are similar to the current microsatellite estimates, across the total dataset (Table 1, S5 Table). These estimates could be ‘artifactually’ inflated by the life history characteristics of crocodiles: a long-lived species with late maturity, and overlapping generations. Crocodile populations in the Okavango Delta that have partially recovered, in absolute population numbers, and have retained genetic diversity, still showed a decline in effective population size [56]. Thus, there is a generational “lagging effect” due to the life history of the species that obscures the true genetic health of such populations. Similarly, a recent survey of the Lower Kunene River and the Kwando River (Namibia) estimated 2.29 crocodiles per kilometre [57], an abundance considered healthy and comparable to the 6.5 and 0.5 crocodiles per kilometre found in the Mahango Game Reserve and the Community River Area within the Okavango Delta, respectively [58]. However, the current estimates for the lower bound of N_e_ for the Lower Kunene River seems to suggest that the population might be vulnerable. In fact, the lower bound estimate for N_e_ for each of the five geographic populations analysed here was lower than the 500–1,000 estimate that is indicates a robust and resilient population [59]. More contentious arguments place these values at higher than 1,000 to ensure long-term evolutionary potential [60]. Notably, the Kunene, Limpopo River and KwaZulu-Natal samples seem to have fairly large confidence intervals, likely as result of the small sample sizes; however, Waples and Do [61] argued that the lower bound estimate might still prove to be a useful indicator. Furthermore, there is evidence to suggest that these populations are contracting, likely due to a ‘recent’ population bottleneck that occurred in the last five generations (Table 3).

Currently, inbreeding (assessed based on F_is_) seems to be low; however, F_is_-values may underestimate the true level of inbreeding when populations have undergone a recent bottleneck. Bottlenecks create a transient inflation of the observed heterozygosity, relative to the expected heterozygosity due to the loss of low frequency alleles, resulting in a lower F_is_ estimate [62]. The mean relatedness within all five population clusters was, however, was significantly higher than zero, which may indicate an incidence of consanguineous mating higher than expected (S3 Fig). The KwaZulu-Natal population, had the highest F_is_ and relatedness coefficients, suggesting that the population might be truly isolated.

## Conclusion

This study contributes with new insights at the geographic fringes of the distribution of the species in southern Africa. Only *C*. *niloticus* was found in the distribution of mitochondrial haplotypes suggesting the existence of different ancestral populations across vast regions of sub-Saharan Africa, most probably resulting from geographical changes in topology. Furthermore, spatial patterns of genetic variation partitioned populations from east and west southern Africa at the upper hierarchical level, with further stratification at the regional level conforming to river system or geographical area. Genetic diversity within populations seemed moderate and comparable to previous studies; however, there was evidence for population contraction with increasing levels of inbreeding. These results provide an increased understanding of Nile crocodile populations in southern Africa, and have utility in conservation and management of this keystone species.

## Supporting information

S1 Fig Broad geographical location of Nile crocodile sampling sites in southern Africa. (PDF)

S2 Fig Summary of STRUCTURE clustering results for the estimation of the most likely number of population clusters. (PDF)

S3 Fig Mean pairwise relatedness coefficients (r) within each population cluster. (PDF)

S1 Table Collection sites of Nile crocodiles in four southern African river systems and genetic markers used in this study. (XLSX)

S2 Table Geographic collection sites of publicly available modern sequences of Nile crocodiles [8] co-analysed in this study and their mitochondrial haplotypes, as determined in this work. (XLSX)

S3 Table Panel of eleven microsatellite markers optimised for cross-species genotyping of Nile crocodiles in three PCR multiplex and one singleplex reactions. T_a_—primer annealing temperature.
(XLSX)

S4 Table Standard genetic diversity measures of Nile crocodile in three river systems in southern Africa. N—number of individuals, H—number of haplotypes, h—haplotype diversity, π - nucleotide diversity, k—mean number of nucleotide differences between haplotypes.
(XLSX)

S5 Table Locus-by-locus and average genetic diversity measures, and HWE test in four southern African river systems (Lower Kunene River, Okavango River and Lower Shire River, and one commercial crocodile farm from Limpopo, South Africa). N—number of individuals, An—number of alleles, Rs—mean allelic richness, He—expected heterozygosity, Ho—observed heterozygosity, Fis—mean Fixation index, HWE—Hardy Weinberg Equilibrium test (*P*-value), Frnull—null allele frequency—Brookfield 1, and PIC—Polymorphic Information Content. *HWE significance after Bonferroni correction for multiple tests (*P* < 0.0045). **Presence of null alleles.
(XLSX)

S6 Table Log Bayes Factor (LBF) using thermodynamic integration of different gene flow models (*Mi*) compared with Model 1 for three sampling regions of *Crocodylus niloticus* within the Okavango River system (OR_OD: Okavango River Delta; OR_OCR: Okavango Crocodile Ranch; OR_BNP: Bwabwatwa National Park; OR_Bot: Botswana; OR_Nam: Namibia); p_*Mi*_−model choice probability; lmL–log marginal likelihood. (XLSX)

S7 Table Log Bayes Factor (LBF) using thermodynamic integration of different gene flow models (*Mi*) compared with Model 3 for two sampling regions of *Crocodylus niloticus* within the Lower Shire system (LS_N: Lower Shire North; LS_S: Lower Shire South); p_*Mi*_−model choice probability; lmL–log marginal likelihood. (XLSX)

S1 Data Supporting data: mtDNA sequence alignment. A 514-bp fragment sequence alignment of the mtDNA control region in nexus format.
(TXT)

S2 Data Supporting data: microsatellite genotypes. Microsatellite genotypes for the study populations in GenePop format.
(NEXUS)
