# Supplementary figures and images for: Phylogeography, genetic diversity, and population structure of Nile crocodile populations at the fringes of the southern African distribution
Source: PLoS One. 2019 Dec 23;14(12):e0226505. doi: 10.1371/journal.pone.0226505 (PMC6927622; doi:10.1371/journal.pone.0226505)

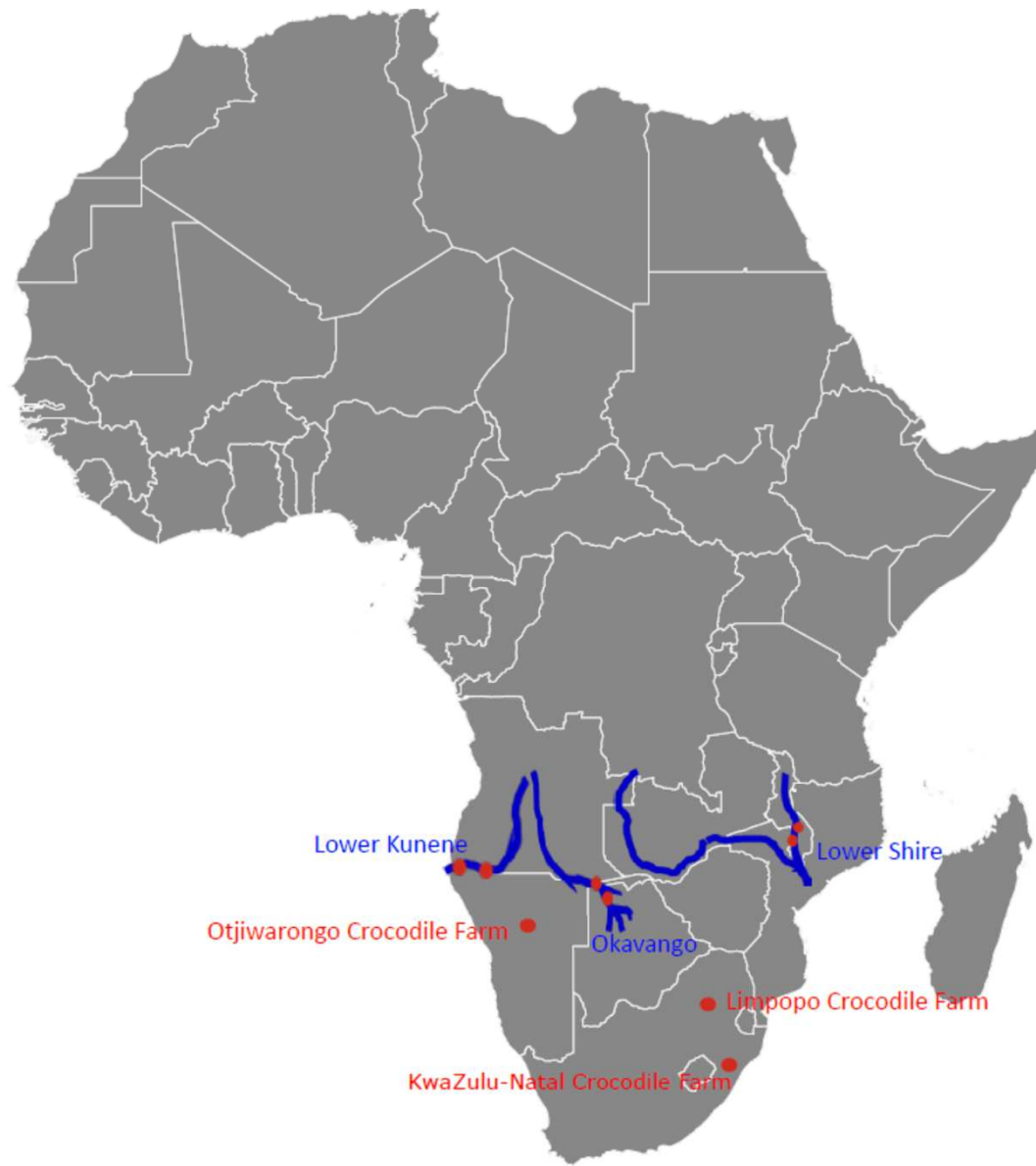

**S1 Fig. Broad geographical location of Nile crocodile sampling sites in southern Africa.**

Supplement: S1 Fig — (PDF) [file pone.0226505.s001.pdf]
