# Supplementary material for: Phylogeography, genetic diversity, and population structure of Nile crocodile populations at the fringes of the southern African distribution
Source: PLoS One. 2019 Dec 23;14(12):e0226505. doi: 10.1371/journal.pone.0226505 (PMC6927622; doi:10.1371/journal.pone.0226505)
Supplement: S2 Fig — (PDF) [file pone.0226505.s002.pdf]

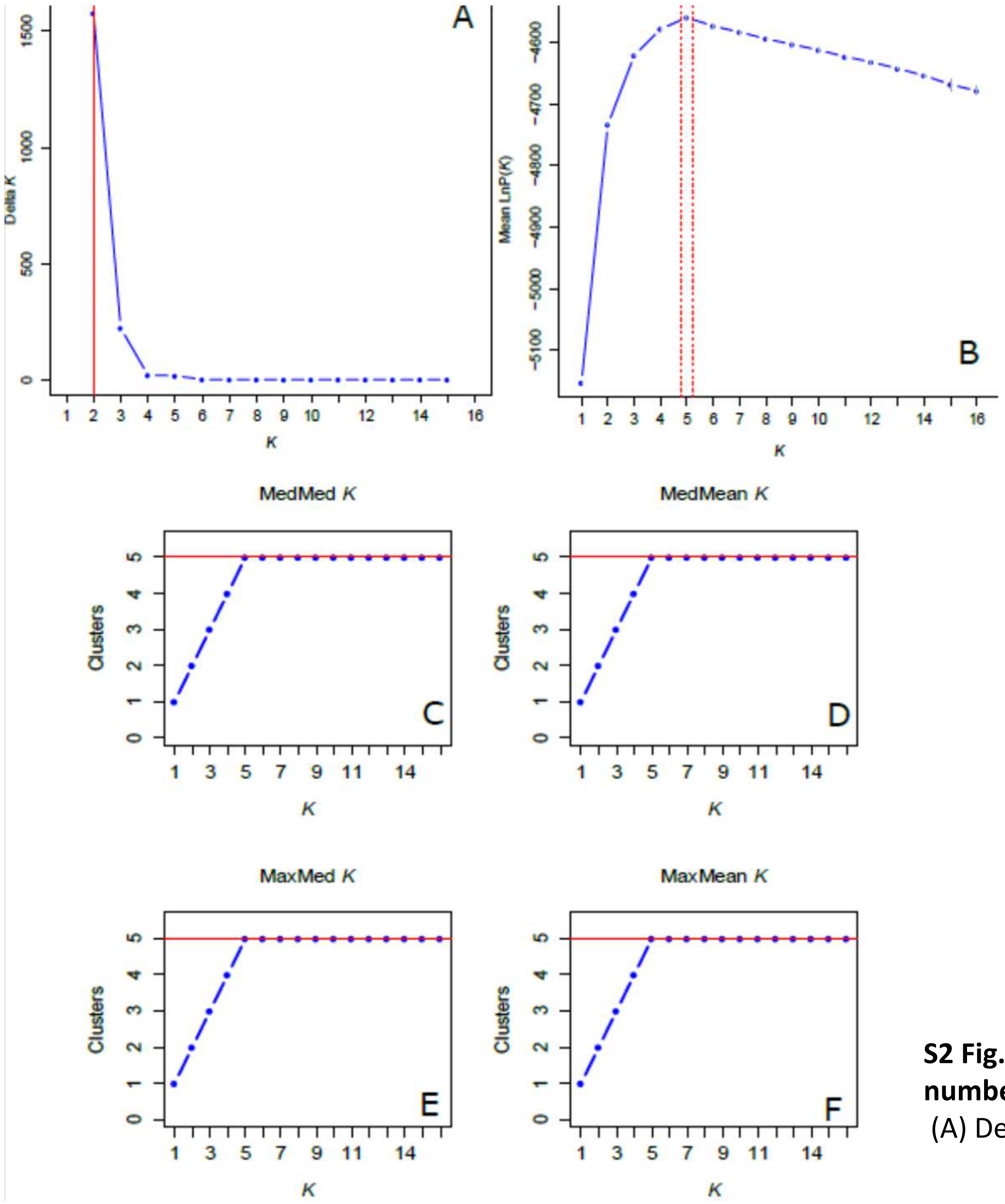

S2 Fig. Summary of STRUCTURE clustering results for the estimation of the most likely number of population clusters.

(A) Delta *K*, (B) Mean LnP*K*, (C) MedMed*K*, (D) MedMean*K*, (E) MaxMed*K*, (F) MaxMean*K*
