# Supplementary material for: Phylogeography, genetic diversity, and population structure of Nile crocodile populations at the fringes of the southern African distribution
Source: PLoS One. 2019 Dec 23;14(12):e0226505. doi: 10.1371/journal.pone.0226505 (PMC6927622; doi:10.1371/journal.pone.0226505)
Supplement: S3 Fig — (PDF) [file pone.0226505.s003.pdf]

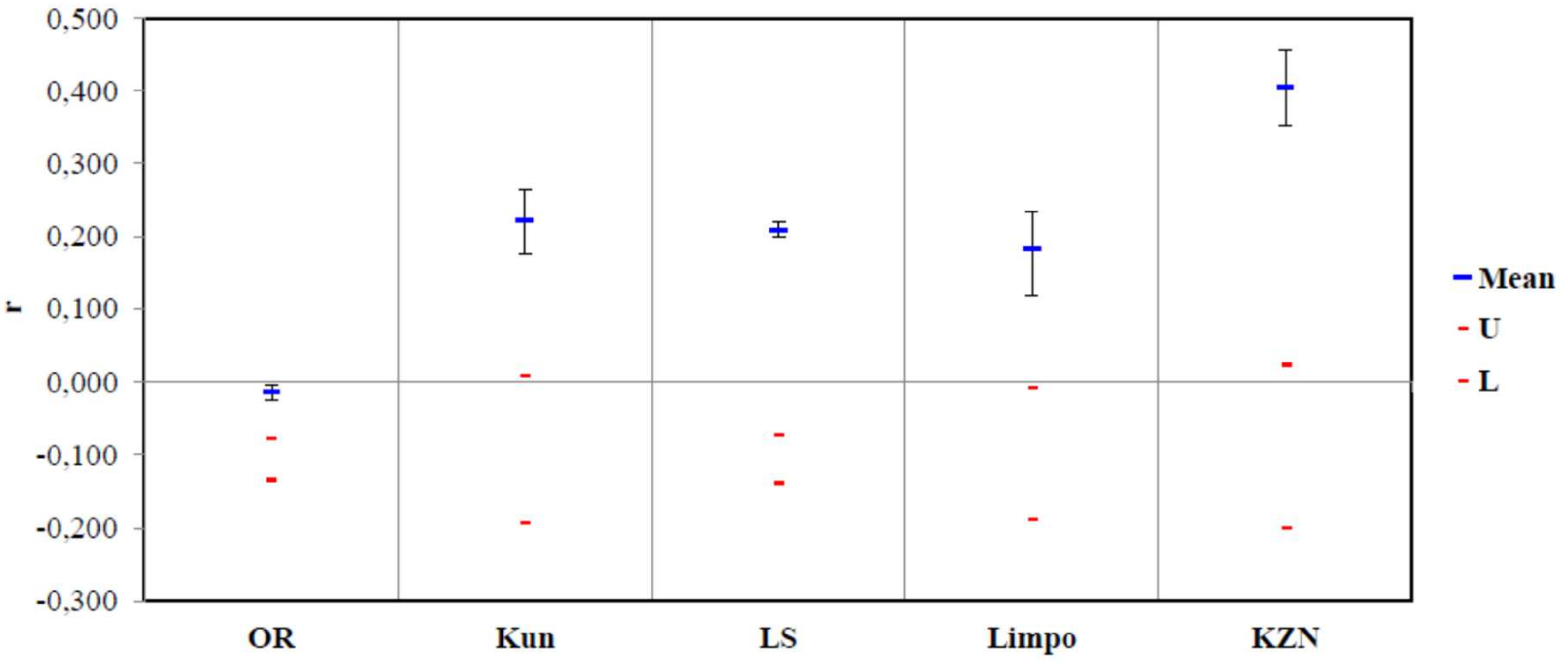

S3 Fig. Mean pairwise relatedness coefficients (r) within each population cluster.
OR (Okavango River), Kun (Kunene River), LS (Lower Shire), Limpo (Limpopo, South Africa), and KZN (KwaZulu-Natal, South Africa). Error bars represent 95% confidence about the means. The Upper (U) and Lower (L) bounds represent 95% CI for significant differences between the means of the population clusters.
